# Integrated microscopy techniques for comprehensive pathology evaluation of an implantable left atrial pressure sensor

**DOI:** 10.1179/2046023613Y.0000000021

**Published:** 2013-03

**Authors:** A Roberts, K E Trainor, B Weeks, N Jackson, R W Troughton, C J Charles, M T Rademaker, I C Melton, I G Crozier, W Hafelfinger, D E Gutfinger, N L Eigler, W T Abraham, F J Clubb

**Affiliations:** 1 Department of Veterinary Pathobiology, Texas A&M University, College Station, TX, USA; 4 Department of Cardiology, Christchurch Hospital and University of Otago, Christchurch, New Zealand; 3 St. Jude Medical, Cardiac Rhythm Management Division, Sylmar, CA, USA; 4 Division of Cardiovascular Medicine, Ohio State University, Columbus, OH, USA

**Keywords:** Histopathology, Left atrial pressure monitor, Medical device evaluation, Medical implant, Micro-computed tomography, Plastic histology, Scanning electron microscopy

## Abstract

The safety and efficacy of an implantable left atrial pressure (LAP) monitoring system is being evaluated in a clinical trial setting. Because the number of available specimens from the clinical trial for histopathology analysis is limited, it is beneficial to maximize the usage of each available specimen by relying on integrated microscopy techniques. The aim of this study is to demonstrate how a comprehensive pathology analysis of a single specimen may be reliably achieved using integrated microscopy techniques. Integrated microscopy techniques consisting of high-resolution gross digital photography followed by micro-computed tomography (micro-CT) scanning, low-vacuum scanning electron microscopy (LVSEM), and microground histology with special stains were applied to the same specimen. Integrated microscopy techniques were applied to eight human specimens. Micro-CT evaluation was beneficial for pinpointing the location and position of the device within the tissue, and for identifying any areas of interest or structural flaws that required additional examination. Usage of LVSEM was reliable in analyzing surface topography and cell type without destroying the integrity of the specimen. Following LVSEM, the specimen remained suitable for embedding in plastic and sectioning for light microscopy, using the positional data gathered from the micro-CT to intersect areas of interest in the slide. Finally, hematoxylin and eosin (H&E) and methylene blue staining was deployed on the slides with high-resolution results. The integration of multiple techniques on a single specimen maximized the usage of the limited number of available specimens from the clinical trial setting. Additionally, this integrated microscopic evaluation approach was found to have the added benefit of providing greater assurance of the derived conclusions because it was possible to cross-validate the results from multiple tests on the same specimen.

## Introduction

Implantable hemodynamic monitoring systems designed to facilitate the management of patients with advanced chronic heart failure have recently been investigated with the aim of reducing hospitalizations for acute decompensated heart failure.^1^–^3^ One such system is an implantable left atrial pressure (LAP) monitor that is linked to a physician-directed, patient self-management treatment paradigm.^3^ Although early clinical studies showed that the LAP monitoring system can be implanted safely into the left atrium,^3^ a close evaluation of the histopathological appearance of the implanted sensor and the host tissue response is critical for demonstrating long-term safety.

Owing to the limited number of specimens available for analysis from the clinical trial setting, it is important to optimally leverage each available specimen. To that end, an approach that integrates a series of complimentary microscopic techniques for pathology evaluation was developed to maximize the quality and quantity of data collected from each specimen, while minimizing the number of required specimens for analysis. A detailed description of this integrated microscopic evaluation approach, along with its application to the analysis of a single human specimen containing the LAP sensor, is presented.

## Materials and Methods

Integrated microscopy techniques consisting of high-resolution gross digital photography followed by micro X-ray, micro-computed tomography (micro-CT) scanning, low-vacuum scanning electron microscopy (LVSEM), and microground plastic-embedded histology were applied to the same specimen. These techniques optimized the evaluation of the integrity of the device, the position of the device and its fixation anchors within the inter-atrial septum, the composition of the tissue surrounding the device, and the overall host response to the presence of the device within the inter-atrial septum.

### Specimens

There were a total of eight histopathology specimens available from patients implanted with the LAP monitoring system in the Hemodynamically Guided Home Self-Therapy in Severe Heart Failure Patients (HOMEOSTASIS) trial.^3^ The implanted portion of the LAP monitoring system (HeartPOD™; St. Jude Medical, CRMD, Sylmar, CA, USA) consists of an implantable sensor lead and coil antenna. The sensor module at the distal end of the implantable sensor lead is implanted into the left atrium via transseptal catheterization and affixed to the inter-atrial septum with proximal and distal folding nitinol anchors. The fixation anchors were designed such that the sensor diaphragm located at the distal tip of the sensor module would protrude approximately 2 mm into the left atrium.

HOMEOSTASIS was a prospective, multicenter, observational, open-label registry, comprising the first human use of the LAP monitoring system for the management of chronic severe heart failure. The trial was approved by the US Food and Drug Administration (IDE G050018) and the appropriate institutional and ethics committee approval was obtained at each center. All subjects gave written informed consent to participate in the trial. For the patients for whom histopathology specimens was available, permission to perform an autopsy and explant the device was also obtained.

### High-resolution digital photography

The LAP sensor module at the implant site, along with a several centimeter radius of adjacent inter-atrial septal tissue, was excised from the heart at the time of autopsy and immersed within a 10% neutral buffered formalin fixation solution. A high-resolution digital single lens reflex camera (Canon Mark 3; Canon Inc., Lake Success, NY, USA) was used to photograph the specimen from multiple perspectives and at multiple magnifications. The photographs were used to aid in determining the specimen orientation within the inter-atrial septum and to identify areas of interest that could subsequently be investigated in greater detail. The photographs also provided a record of the gross morphology of the tissue surrounding the device.

### Micro-CT and micro X-ray

The specimen was scanned using a HAWK-160XI (Nikon Metrology Inc., Brighton, MI, USA) micro-CT system. The system uses a cone-beam X-ray detector and source configuration for image acquisition with a tungsten target for generating X-rays up to 160 kV. Approximately 1000 projections were completed for each scan, with an average of two scans performed on each specimen. The scanning process was carried out with Inspect-X software (Nikon Metrology Inc.), and volumes were reconstructed with CT-Pro (Nikon Metrology Inc.). Micro-CT data were analyzed and reconstructed in three dimensions in VGStudioMAX (Volume Graphics GmbH, Heidelberg, Germany). Device orientation within the inter-atrial septum, its structural integrity, and other information pertaining to the functioning of the device was assessed based on the three dimensional reconstruction data and used to identify areas of interest for more detailed analysis.

Two dimensional traditional X-ray micrographs were also produced using the same equipment described above. X-ray radiographs were taken in both *en face* and lateral orientations, at a lower power than CT (at 120kV, 11 μA) that enabled the resolution of the soft tissue surrounding the device.

### LVSEM

Specimens submitted for low vacuum SEM were processed *in toto* in graded ethyl alcohol (stepping from 10% to absolute in 10% increments), desiccated in a vacuum desiccator for 5 minutes, and placed in the JEOL SEM scope (JSM-6460LV; JEOL, Tokyo, Japan). After imaging, the specimens were rehydrated and post-fixed in 10% neutral buffered formalin or other fixatives as necessary.

### Plastic embedding and histology

Study specimens and surrounding tissue were embedded in isobornyl methacrylate plastic resin (Technovit 7200; Heraeus Kulzer, Wehrheim, Germany). The target site along the center of the device, perpendicular to the long axis, was located using measurements taken from micro-CT data in order to orient the sectioning equipment and block. Then the plastic-embedded specimens were sectioned in slices along the long axis of the sensor module using a diamond-studded wire saw (Exakt Technologies Inc., Oklahoma City, OK, USA). The sections were glued to slides, ground using an automated grinder (Exakt Technologies Inc.) to 50–75 μm thick, and polished.^4^

Slides were stained as follows: (1) slides were etched for 2 minutes in 2% formic acid solution (vol/vol); (2) 5 minutes in 50% ethanol (vol/vol); (3) on a hot plate set to 55°C, a bead of Gill’s #3 hematoxylin (Electron Microscopy Sciences, Hatfield, PA, USA) was placed over the specimen, and allowed to stain for 35 minutes; (4) Slides were rinsed in distilled water and stained in a 50%/50% mixture of Eosin Y (Electron Microscopy Sciences) and Phyloxine B (Electron Microscopy Sciences) (vol/vol); and (5) rinsed in distilled water and cleared in 50% ethanol (vol/vol) with 2–3 drops of acetic acid (Electron Microscopy Sciences). The slides were left without a coverslip and examined microscopically under a small amount of immersion oil. Additional slides were stained with Methylene Blue (Electron Microscopy Sciences) for 10 minutes to visualize collagenous connective tissue and photographed in the same manner. The microscopic evaluation was directed toward determining the extent of tissue coverage, cell type covering the sensor module, degree of inflammation, and the presence of any thrombosis.

## Results

All eight human specimens of the LAP sensor were successfully processed using integrated microscopy techniques. All eight specimens demonstrated that the LAP sensor had neoendocardial tissue overgrowth with no excessive host-to-device reaction when implanted through the inter-atrial septum for durations ranging between 3 months and greater than 4½ years. An illustrative case example is presented for the results obtained from a single specimen of the LAP sensor that was implanted for a period of 1686 days in a 74-year-old gentleman with an ischemic cardiomyopathy.

### High-resolution digital photography

**Figure 1:**
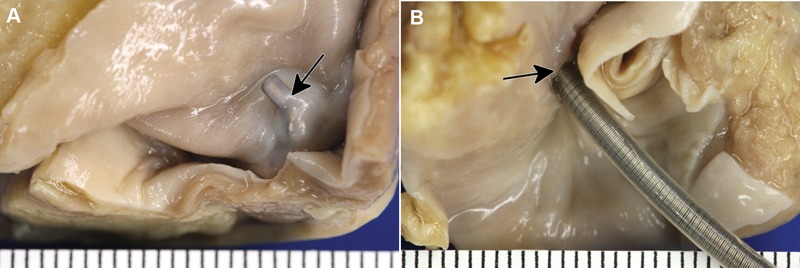
Gross photography. (A) The view from the left side of the inter-atrial septum shows neoendocardium covering the sensor diaphragm (indicated by an arrow) with an orthogonal placement of the sensor module relative to the plane of the inter-atrial septum (ruler with 1 mm ticks). (B) The view from the right side of the inter-atrial septum shows minimal changes and a circumferential covering of connective tissue (indicated by an arrow) at the base (ruler with 1 mm ticks).

Figure 1 shows high-resolution digital photographs of the formalin-fixed gross specimen submitted for evaluation. The photograph shows that the sensor module is oriented close to perpendicular to the plane of the inter-atrial septum, and that the sensor diaphragm is covered with a relatively thick layer of connective tissue with no evidence of thrombus (Fig. 1A). Gross examination from the right side of the inter-atrial septum (Fig. 1B) shows the presence of a thin circumferential covering of connective tissue at the base of the sensor module, that tapers to the lead surface over a distance of approximately 2 mm from the inter-atrial septum.

### Micro-CT and micro X-ray

**Figure 2:**
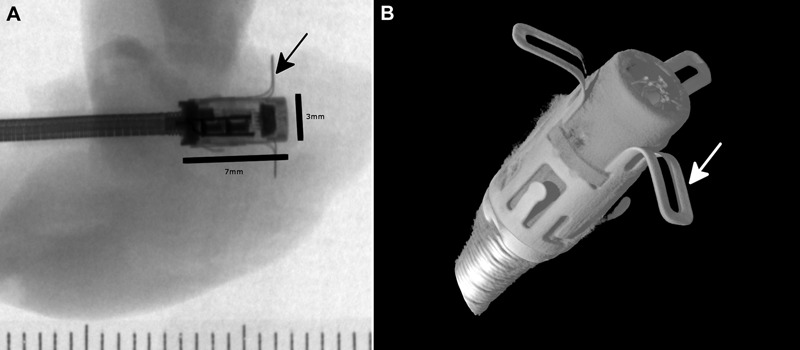
Micro X-ray and micro-CT. (A) Micro X-ray radiograph showing the orthogonal orientation of the sensor module within the inter-atrial septum, with no evidence of damage or disruption to the external (arrow indicating nitinol anchor) or internal components (ruler with 1 mm short ticks). (B) Micro-CT reconstruction showing no damage to the fixation anchors (indicated by an arrow).

Micro X-ray images of the device show no evidence of any internal or external defects to the LAP sensor module (Fig. 2A). In particular, there was no evidence of a fracture of the nitinol anchors. Three-dimensional reconstructions from the micro-CT scan validated these findings and allowed high-resolution visualization of the device for qualitative analysis purposes, both externally and internally, along various cross-sections and orientations (Fig. 2B).

### LVSEM

**Figure 3:**
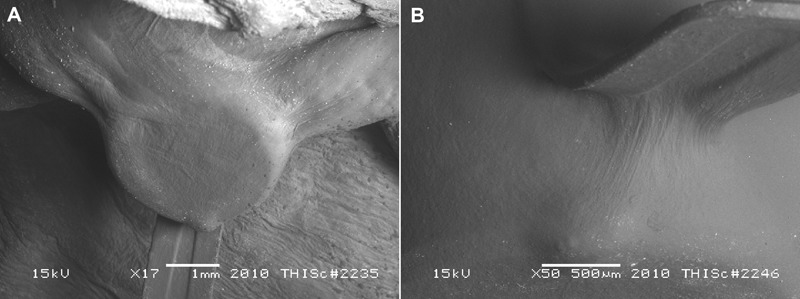
Low-vacuum scanning electron microscopy. (A) The neoendocardium covering the sensor diaphragm and the distal anchors can be seen. The neoendocardium appears to be smooth and continuous with the anchors and the endocardium (scale bar = 1 mm). (B) A continuous layer of smooth neoendocardium can be seen filling the gap between the loop of the anchor (scale bar = 500 *μ*m).

Figure 3 shows the LVSEM images obtained from the same specimen shown in Fig. 1. Despite the low-vacuum scanner being slightly less powerful than its high-vacuum counterpart, the micrographs obtained were able to resolve endothelial cell growth, over the surface of the device, that protruded into the left atrial chamber. A very thin layer of smooth endothelium can be seen covering the distal anchor that is directed toward six o’clock (Fig. 3A). In addition, a thin connective tissue substrate can be seen bridging the space between the loops of this anchor (Fig. 3B). There is evidence of minor linearly-directed, endothelial roughening on the covering of the sensor diaphragm and isolated endothelial cell sloughing (bionecrosis). There is also minimal evidence of platelet aggregation that can be seen on the tissue substrate.

### Plastic embedding and histology

**Figure 4:**
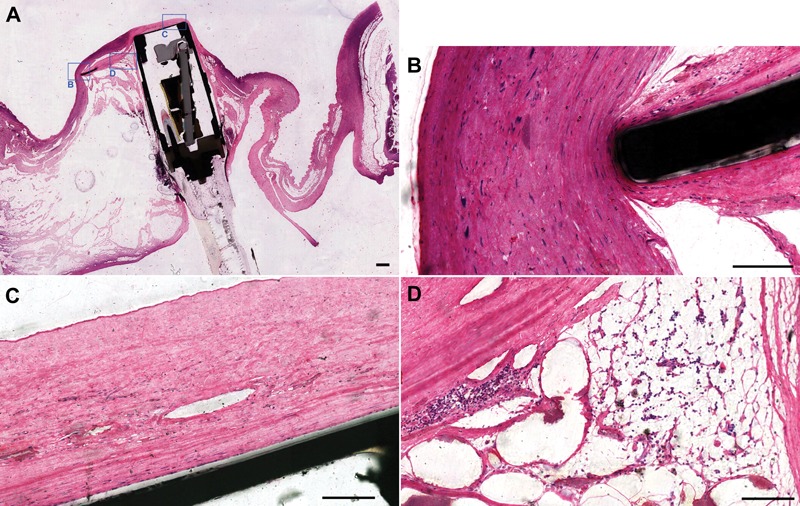
Plastic histology. (A) Subgross (original magnification: ×1) image of the middle transverse section of the sensor module (scale bar = 100 mm). (B) Tip of the distal anchor showing neoendocardium growth over the tip of the distal anchor (original magnification: ×30, scale bar = 100 *μ*m). (C) Tissue capsule covering the sensor diaphragm, showing small blood vessels, layers of fibrin, and evidence of enmeshed erythrocytes (original magnification: ×32, scale bar = 100 *μ*m). (D) Pericapsular region showing adipose cell metaplasia at the interface between the sensor module and the myocardium with admixed inflammatory cells and macrophages, some hemosiderin-laden (original magnification: ×35, scale bar = 100 *μ*m).

A total of three longitudinal sections were typically obtained from each specimen. The midline section (Fig. 4A) shows the transseptal placement of the device. Closer inspection of the distal anchor (Fig. 4B) showed fibrous connective tissue and neoendocardium completely covering the tip of the distal anchor (varying from 0·25 to 0·8 mm in thickness). Because the distal anchors rest flush against the surface of the inter-atrial septum, the distal anchors commonly trigger the formation of a fibrous connective tissue capsule that is lined with neoendocardium. The sensor diaphragm can be seen extending approximately 3·2 mm into the left atrium relative to the membranous part of the inter-atrial septum. The blood-contacting surface of the sensor diaphragm (Fig. 4C) is lined with endothelium and covered by an approximately 0·36-mm-thick cellular connective tissue layer (consisting of myofibroblasts, blood vessels, and extravasated erythrocytes). Within the pericapsule substrate (Fig. 4D), low numbers of multinucleated giant cells admixed with scattered infiltrates of macrophages (some hemosiderin-laden) can be seen. These are expected cellular inflammatory findings following foreign body penetration into tissue, which in this case was the implanted medical device. This interface is expanded on the myocardial side by a roughly triangular-shaped area consisting of pre-existing myocardial degeneration with fibrosis, plump fibroblasts, and adipose cell metaplasia. These are typical microscopic hallmarks of the healing response to the implanted medical device. The trans-septal connective tissue capsule surrounding the sensor module varies from 0·3 to 0·75 mm in thickness. The blood-contacting surfaces on both the right and left sides of the inter-atrial septum were found to be covered by endocardium (Fig. 4A). The insertion site at both entrance and exit of the inter-atrial septum shows a mild to moderate connective tissue interface with myocardium. There is no microscopic evidence of active mural thrombosis.

## Discussion

The pre-clinical and clinical testing phases for biomedical devices demand comprehensive scrutiny. Full-fledged investigations can become both expensive and time-consuming due to the demand of multiple samples that must be used in various testing methodologies to obtain data on the safety and performance of the device. This manuscript demonstrates how a comprehensive pathology evaluation of a single specimen of the LAP sensor may be achieved reliably using an integrated approach leveraging a series of microscopy techniques. The applicability of this approach to other medical devices, such as intravascular stents, has already been demonstrated by Timmons and coworkers^5^ However, the Timmons' and colleagues manuscript did not incorporate the usage of LVSEM.

Four complimentary microscopy techniques consisting of high resolution digital photography followed by micro-CT scanning, LVSEM, and microground histology were utilized sequentially to process each specimen. Digital photography, micro-CT scanning, and LVSEM are performed before microground histology because there are non-destructive. High-resolution digital photography provides images of the orientation of the device within the tissues and of the gross pathology. Micro-CT scanning makes it possible to visualize the external and internal components of the device non-invasively, with the ability to reconstruct images of the device using multiple projections and orientations. Micro-CT scanning is valuable in examining the structural integrity of the device, and in guiding the micro-sectioning of the device. LVSEM supplements the histopathological evaluation by providing high-magnification images of the surface of the device and surrounding tissues that allows the identification of endothelial cells, fibrin, platelets, and other cellular deposits. If desired, LVSEM may be performed before micro-CT scanning, as an alternative approach. The microground histology provides subgross and microscopic pathology images of the specimen that, when combined with the LVSEM, aid in the interpretation of the gross pathology. Since for each specimen, there are both LVSEM and microground histologies available, it becomes possible to determine for each specimen the host-to-device reaction with greater confidence.

### Micro-CT

Micro-CT has been proven to be a valuable technology for evaluating medical devices.^6^ The capability to identify structural defects or complications related to the performance of the device, as well as the ability to obtain positional data for precise sectioning allows a more controlled and an efficient approach to device evaluation. Micro-CT scanning also provides a low-cost alternative to traditional device performance diagnostics by producing a three-dimensional image of the current state of the components of the device.^7^ This micro-imaging approach for evaluating miniature devices could reduce the turnaround time during the research and development phases of the device in cases that do not require histology or further analysis with other methods.

### LVSEM

Standard scanning electron microscopes employ a high-vacuum specimen chamber that is not tolerant of water vapor or other gaseous materials. A biological specimen that is to be examined in a high-vacuum SEM must be prepared by dehydration, usually critical point drying, and sputter coating. In this procedure, the surface is coated with a conductive material that stabilizes it in the vacuum and also serves as a conductive conduit for the charge from the electron beam to dissipate.^8^ However, recent developments in SEM technology have produced microscopes that do not require a high-vacuum preparation. A LVSEM, also sometimes referred to as an environmental scanning electron microscope, can image a tissue specimen that has been dehydrated using graded alcohols in a chamber evacuated to 1–10 torr (0.133 kPa–1·3 kPa). This is accomplished by isolating the different apertures in different chambers, and only evacuating the chambers that must be held at high vacuum.^9^

The rehydration and fixation of tissue post-LVSEM allows the same specimen to be submitted for further examination. By avoiding the gold coating and high-vacuum components of traditional SEM tissue preparation, the artifact damage sometimes associated with those procedures is avoided.

### Plastic embedding and histology

The use of paraffin histology slides is not applicable for tissue that contains medical devices such as the LAP sensor, because of difficulties encountered when attempting to section the paraffin tissue block. To overcome this limitation, plastic-embedded histology is needed, which results in histology sections that tend to be thicker compared to the traditional paraffin slides.

One of the most commonly used resins for plastic-embedded histology is glycol methacrylate, because it is sufficiently soft and easy to cut and stain using the standard techniques used to process paraffin.^10^,^11^ This is in contrast to isobornyl methacrylate (also known as Technovit 7200), which has a much denser substrate that is more brittle and cannot be removed in order to stain the tissue without specialized techniques. Because of the brittle nature of Technovit 7200, it cannot be cut on a microtome and requires specialized sawing and grinding equipment in order to produce sufficiently thin sections. Despite these limitations, the usage of Technovit 7200 is favored because the tissue shrinkage associated with processing is minimal compared to other techniques, and because the hardness of the resin prevents distortion of the tissue/metal interface during processing. These two factors taken together have the benefit of producing very high quality, low artifact sections with minimal tissue distortion that allow for precise morphological measurements that may be used to cross-validate measurements taken from other techniques.^5^

Early research addressing the feasibility of staining tissues embedded within dense methacrylate plastics with traditional hematoxylin and eosin (H&E) produced poor results,^4^,^12^ attributed to poor tissue penetration. Subsequently, smaller molecular weight dyes that are capable of penetrating the plastic matrix more easily were used and yielded better results. Celestine blue commonly substitutes as the basic dye, while acid fuchsin serves as the acidic counterstain.^13^ These substitutes commonly produce a final result very similar to traditional H&E when used on thin plastic sections in the range of 10–40 μm, because the small molecule stains can penetrate the entire section and provide sufficient contrast. However, it is difficult to routinely achieve sectioning thicknesses in this range without risking the potential loss of the specimen, which is disastrous when the number of available specimens is limited.

To circumvent these limitations, an approach was developed that provided increased versatility by using thicker sections (50–75 μm) and relying on formic acid etching, ethanol dehydration, and heat in order to facilitate the penetration of Gill’s #3 hematoxylin molecules, which have a large molecular weight, into the plastic matrix. These plastic processing techniques have been applied separately in the past on the smaller molecular weight dyes to enhance stain penetration and contrast,^14^,^15^ but when they are combined they are just as effective for larger molecule stains as well. This improved protocol was developed for the diagnostic capability and familiarity of the traditional H&E stain for analyses. The specificity for organelles and cellular structures of Gill’s #3 hematoxylin molecules resulted in a high quality and a fine detailed visualization of the microscopic structures in plastic.

Using this technique, larger Gill’s #3 Hematoxylin molecules can penetrate the upper surface of the slide and stain the tissue through what appear to be micro-cracks and pores that are formed as a result of the formic acid etching process. When the section is polished after staining, and roughly 10 μm of material is removed, the stain is no longer visible because the stain only extends 10 μm into the surface of the plastic section. Afterwards, the section can be re-stained with the same procedure to produce the same results, or a different stain if desired. When compared to the traditional techniques that focus on staining the entire volume of tissue on the slide, the overwhelming contrast from the thick section staining and the difficulties in visualizing cellular components is avoided because the stain only affects the top 10 μm of the slide. Additionally, sectioning methods using the small weight dye stains demand that the section be as thin as possible in order to provide resolution of small tissue structures without overwhelming contrast from a thick section.^14^ The etching technique does not have these problems because a thin enough portion of the material is stained to provide resolution. Finally, since the dye molecules are more specific to cellular structures, there are no problems with contrast or background staining.

## Conclusion

The integration of multiple techniques on a single specimen maximized the usage of the limited number of available specimens. Micro-CT analysis allowed precise measurements of the orientation of the device within the tissue and helped identify areas of interest or structural flaws that required additional examination. The LVSEM system was capable of analyzing surface topography and endothelial proliferation without compromising the integrity of the specimen. Following LVSEM, the specimen was suitable for embedding in plastic and sectioning for light microscopy using the orientation data gathered from the Micro-CT analysis. Finally, by modifying and combining procedures to treat the plastic-substrate before staining, it was possible to visualize high details at the cellular level that previously could not be seen using other approaches for plastic embedded histology. Detailed evaluation of the cellular healing response to the implanted medical device, such as that described in this manuscript, is valuable for demonstrating long-term safety.
